# Hidden reservoirs of pathogens in dental settings

**DOI:** 10.6026/97320630017073

**Published:** 2021-01-31

**Authors:** Silpi Chatterjee, Sonal Saigal, Ankur Bhargava, Daya Shankar, Asim Mustafa Khan, Safiya Fatima Khan

**Affiliations:** 1 Department of Public Health Dentistry, Hazaribag college of Dental Sciences and Hospital, Jharkhand, India; 2 Department of Oral Pathology and microbiology and forensic odontology, Dental Institute, RIMS, Ranchi, India; 3 Department of Oral Pathology and microbiology and forensic odontology, Hazaribag college of Dental Sciences and Hospital, Jharkhand, India; 4 Department of rosthodontics and Crwon & Bridge. Hazaribag college of Dental Sciences and Hospital, Jharkhand, India; 5 Department of Biomedical Dental Sciences Imam Abdulrahman Bin Faisal University, Saudi Arabia; 6 Department of Periodontology Faculty of Dental Sciences, Ramaiah University of Applied Sciences, India

**Keywords:** Disinfection, infection, microbial contamination, pathogenic organism

## Abstract

Nosocomial infections are a major concern to both clinicians and health care seekers. Investigations have suggested that laptops & mobile phones may contribute to cross-contamination and can serve as vehicles for infection transmission. Therefore, it is of interest to document the data on hidden reservoirs such as mobile phones and laptops of pathogens in dental settings at the Hazaribag College of dental sciences and Hospital, Jharkhand. The samples were collected from 25 laptops and 25 mobile phones from dentists working in a dental college in Hazaribag city. The samples were collected aseptically using sterile cotton swabs dipped in sterile saline by rotating the swabs on the keyboard surfaces of laptops and mobile phones, inoculated into Brain Heart Infusion broth, vortexed for 1 minute in Fischer Vortex Genie 2 on highest setting & streaked immediately on 5% sheep blood agar plates and were incubated at 37°C for 24 hours aerobically. The isolates were identified based on the colony morphology, colony characteristics and biochemical reactions. The bacterial species isolated were Staphylococcus aureus, Coagulase negative Staphylococcus, Bacillus species, Enterococci, Micrococci, and Pseudomonas etc. Predominant species isolated was Staphylococcus aureus and least was Micrococci. Higher percentage of organisms was found at the Department of Periodontics, Endodontics and least was found in Department of Public Health Dentistry. The percentage and type of organism isolated from keyboards of laptops and mobile phones were similar. Thus, laptops and mobile phones act as vehicles for transfer of potential pathogens associated with dental hospitals. Disinfecting the hands prior to examination of patients and disinfection of laptops and mobiles with alcohol wipes should be done to prevent nosocomial infections.

## Background

Healthcare-associated infections are an important cause of morbidity and mortality in hospitals. Each year more than 2 million patients acquire healthcare-associated infections, resulting in 90,000 deaths and healthcare costs that are estimated to exceed $5 billion. Health care-associated infection (HCAI), also referred to as "nosocomial" or "hospital" infection, is defined as: "An infection occurring in a patient during the process of care in a health-care facility which was not present or incubating at the time of admission"[1]. Some studies have demonstrated that the mean rate of compliance with the Centers for Disease Control and Prevention guidelines on hand hygiene is approximately 40% among healthcare workers [2], which is a likely explanation for the frequent contamination of computer keyboards and mobile phones. With the advent of technology, mobile phones, laptops used by health care professionals are on the rise especially in the clinical set ups. The laptops and mobile phones of health care workers harbor many harmful pathogens which serve as a reservoir for nosocomial infections and may contribute to cross - contamination, which serve as vehicles for infection transmission [3-6]. Studies have revealed that mobile phones and laptops have a great potential for dissemination of disease and the incidence of such cross contamination diseases to be 4.8% in U S A, 7.1% in European countries, 10-30% in India and 17.1 % in Iran [7-10]. Some investigators have suggested that computer keyboards may contribute to cross-transmission because of acquisition of transient hand carriage by healthcare personnel during contact with the contaminated computer keyboard surface [11,12]. Technical support systems have acted as a boon for health care providers in the past few decades. The burden of data recording, data maintenance & analysis of data have become very easy with the introduction of multiple softwares in health care sector. The usage of these has been very simple & can be operated through laptops & mobile phones. This in turns acts as reservoir for health care associated infections. Since laptops and mobile phones have become an essential means of communication, their usage in clinical set up is unavoidable [13]. As mobile phones act as perfect habitat for microbes to breed, especially in high temperature and humid conditions, Health care workers (HCWs') mobile phones may serve as reservoirs of microorganisms that could be easily transmitted from the mobile phones to the HCWs' hands and therefore facilitate the transmission of bacterial isolates from one patient to another in different hospital wards [14]. Dental clinics are commonplace for the bacterial aerosols generated by high-speed dental hand pieces with water supplies, which has the capacity to settle over long distance. Aerosols and spatter produced during many dental procedures are a potential source of transmission of various diseases [15,17]. The use of laptops, desktops, and mobile phones has become an integral part of dental practice. Therefore, it is of interest to document the data on hidden reservoirs such as mobile phones and laptops of pathogens in dental settings at the Hazaribag College of dental sciences and Hospital, Jharkhand.

## Methodology

### Study location:

A cross sectional study was done to assess the microbial contamination of laptops and mobile phones used by dentists in clinical settings of a dental college in Hazaribag city, Jharkhand, India.

### Study Period:

The duration of the study was for a period of 3 months from January 1st to March 31st 2020.

### Ethical Clearance:

Ethical clearance was obtained from the Institutional Ethical Committee before the start of the study. Necessary permission was obtained from the institution prior to the study. Informed consent was obtained from the dentists before the start of the study.

### Study Criteria:

Inclusion criteria were the laptops and mobile phones, which were in use for a minimum period of one year near clinical settings, were taken for the study.

### Consent:

Dentists who did not give consent to participate were excluded from the study.

### Data size:

A pilot study was conducted by collecting the samples from 5 participants.

### Model data:

A sample of 25 laptops and 25 mobile phones, which satisfied eligibility criteria, were considered for the study. The laptops and mobile phones were randomly selected using a simple random sampling technique.

### Microbial analysis:

The samples were collected aseptically using sterile cotton swabs dipped in sterile saline by rotating the swabs on the keys of laptops and mobile phones during operating hours using a method in which the investigator had received training in advance. The swabs were then transported immediately to the laboratory for inoculation. The samples were then inoculated into Brain Heart Infusion (BHI) broth.The sample was vortexed for 1 minute in Fischer vortex genie 2 on highest setting. The samples were then streaked immediately on 5% sheep blood agar plates and were incubated at 370C for 24 hours aerobically. The organisms isolated were stained and identified based on themorphology(shape, arrangement of the organisms), colony characteristics (size, shape of the colony, opacity, pigmentation, haemolysis, elevation etc.) and biochemical reactions(catalase test, coagulase test, sugar fermentation, heat test, citrate utilization test, urease test, triple sugar iron test, oxidase, mannitol motility test etc). The colonies were counted and colony-forming unit was estimated.

### Statistical analysis:

The data analysis was done using the statistical software SPSS version 23. Descriptive statistics was done for the colony forming units and microbial organisms present on laptops and mobile phones of various departments. Pearson correlation was computed for comparing the microbial contamination of laptops & mobile phones with respect to various departments.

## Results:

The organisms isolated were Staphylococcus aureus, Coagulase negative Staphylococci, Micrococci, Enterococci, Diphtheroids, Bacillus anthracis, Bacillus subtilis, Acinetobacter species and Pseudomonas species. Out of 25 laptops, 16.66% of laptops from the Department of Endodontics, 14.76% from Department of Orthodontics, 14.6% from Department of Oral surgery were contaminated. Among mobiles, 25.48% of mobiles from the Department of Periodontics, 24.29% from Orthodontics, 18.51% from Oral surgery were contaminated (Table 1 - see PDF). Staphylococcus aureus was present in 29.2% of mobiles from Periodontics, 20.8% from Orthodontics and 16.7% from Endodontics and 16.6% from Oral surgery. Coagulase negative Staphylococcuswas present in 29.63% of mobiles from Public Health Dentistry, 17.28% from Prosthodontics and 12.3% from Oral medicine (Table 2 - see PDF). Staphylococcus aureus was present in 22.5% of laptops from Endodontics followed by 21.87% from Orthodontics, 18.75% from Oral surgery and 12.5% from Prosthodontics. Coagulase negative Staphylococcuswas present in 25.6% from Endodontics, 19.23% from Orthodontics, 16.67% from Prosthodontics and 11.54% from Oral medicine (Table 2 - see PDF). Staphylococcus aureuswas present in all the laptops (88.89%) and (77.78%) of mobiles (Table 3 - see PDF). Statistically significant and positive correlation was obtained for department of Prosthodontics (r=0.809), Oral pathology (r=0.894) and Endodontics (r=0.860) (Table 4 - see PDF).

## Discussion:

This study shows that a proportion of around two third of all the laptops and mobile phones near clinical setup and almost half of those sampled immediately after use were contaminated with microorganisms, which can lead to nosocomial infections. The microbial contamination was more for the departments of Orthodontics (18.08%) followed by Oral surgery (15.96%) and least was from Endodontics (14.57%). The use of mobile phones and laptops by the dental faculty and postgraduate students involved in direct patient care not only demonstrated a high contamination rate with bacteria but were contaminated with nosocomial pathogens. The organisms isolated were Staphylococcus aureus, Micrococci, Acinetobacter, Bacillus species, Enterococci and Pseudomonas. Among these Staphylococcus aures and Acinetobacter are resistant to drying and can survive for weeks in a dry environment and is capable of multiplying rapidly. Acinetobacter was identified based on Gram stain, oxidase and motility tests. The microbial contamination in the present study among laptops was Staphylococcus aureus25% from department of Periodontics followed by 22.5% from the department of Endodontics, coagulase negative Staphylococcuswas 25.6% from the department of Endodontics which is contradictory to study on laptops which showed 88% of contamination with coagulase negativestaphylococcus and 12% of contamination with staphylococcus aureus [17]. The overall rate of contamination of laptops with potentially pathogenic organisms like Acinetobacter was 62.50% which is similar to a study by William A et al [18] where multidrug resistant Acinetobacter baumannii was found on the hands, cell phones of health care workers and patients admitted to the ICU (60%) and contradictory to a study by Sweta Singh et al. [19] on cell phones which showed lower rates of contamination ranging from 7-14.3%. The higher rates of contamination of laptops and mobile phones among departments in this present study might be due to the influence of various factors like lack of hand washing after examination or treatments, disinfection practices followed in the hospital, frequency of use of gadgets and the frequency of disinfection of laptops and mobile phones. This study showed that 88% of laptops and 98% of the mobile phones were contaminated with more than one pathogenic organism which is similar to a study done by Brady et al. [4] showed that 89.7% of mobile phones were contaminated. The most dominant organism isolated was Staphylococcus aureus.Jesle et al. [20] found that rate of bacterial contamination of hospital care workers (HCW's) was 95% while that of mobile phones was 90% which is similar to a study by Sweta Singh et al. [19] who reported that out of 50 mobile phones cultured, 98% were positive. The present study is contradictory to a study by Harish Trivedi et al [21] where 58.66% of hand samples and 46.66% of mobile phones were contaminated by bacteria. Ulger et al. [22] showed that 94.5% of phones showed evidence of bacterial contamination. They found that 49% of phones had one bacterial species, 34% had two different species and 11.5% had two or more different species which is contradictory to the present study were 20% had single species (n=3), 45% had two species (n=15) and 35% had more than two types of species (n=7). However, lower rates were observed by Ramesh et al. [23] in which, 45% of mobile phones were contaminated. A study by Lu et al. [24] revealed a 17.4% contamination rate of computer devices by Staphylococcus aureus, Acinetobacter species or Pseudomonas species and contradictory to a study by William et al. [18] who studied the degree of microbial contamination of computers, the efficacy of different disinfectants, and the cosmetic and function effects of these disinfectants on computer keyboards. Potential pathogenic microorganisms were cultured from more than 50 percent of the computers. 10.15% of laptops from Department of Periodontics were contaminated followed by 13.96% from Prosthodontics, 16.6% from Endodontics. In case of mobile phones, 25.48% from Periodontics, 10.66% from Endodontics were contaminated, 7.55% from Prosthodontics which is contrast to a study by Sham.S.Bhat et al. [25 where 4 % from Prosthodontics, 5% of mobiles from Orthodontics showed pathogenic organisms. Hence, in a country like India, mobile phones and laptops of HCWs plays an important role in transmission of infection to patients, which can increase the burden of heath care. Simple measures such as increasing hand hygiene and regular decontamination of mobile phones with alcohol disinfectant wipes may reduce the risk of cross contamination caused by these devices. One study reported the use of 70 % isopropyl alcohol as an effective disinfectant15. Another study reported that restricted use of mobile phones during working hours along with proper hand hygiene practices enabled mobile phones to remain free of contamination16. The findings of the present study are alarming which shows that dentists are lacking the awareness of the safety measures when a significant number of them neither clean their hands before and after seeing a patient nor disinfect their laptops and mobile phones after using in the hospital setup. Hand washing is the simplest and most economical measure that can prevent the transfer of harmful pathogens. There are no rules restricting dentists to use laptops and mobile phones into a sterile clinical setup in India. There are also no cleaning guidelines for laptops and mobile phones of health care workers. The design of this study being a cross sectional one doesnot permits causal inference between microorganisms present in laptops and mobiles. Further studies for the assessment of microbial contamination among dental specialties and methods of decontamination of laptops and mobile phones should be formulated.

## Conclusion

Laptops and mobile phones are reservoir of microorganisms associated with healthcare associated infections (HAI). Data shows that 88% of laptops and 98% of the mobile phones were contaminated. It appears that routine disinfection of mobile phones and laptops will be effective in reducing microbial contamination.
